# The role of pulmonary metastasectomy in patients suffering pancreatic ductal adenocarcinoma with lung metastases: a systematic review and meta-analysis

**DOI:** 10.3389/fsurg.2025.1535212

**Published:** 2025-02-27

**Authors:** Pengcheng Zhao, Qiaoqi Jiang, Kang Xue, Xiaofeng Liu, Bole Tian

**Affiliations:** ^1^ Key Laboratory of Birth Defects and Related Diseases of Women and Children (Sichuan University), Ministry of Education, West China Second Hospital, Sichuan University, Chengdu, Sichuan, China; ^2^ Department of Pediatric Surgery, West China Second Hospital, Sichuan University, Chengdu, Sichuan, China; ^3^ Department of Anesthesiology, West China Xiamen Hospital, Sichuan University, Xiamen, Fujian, China; ^4^ Department of General Surgery, Division of Pancreatic Surgery, West China Hospital, Sichuan University, Chengdu, Sichuan, China

**Keywords:** pancreatic ductal adenocarcinoma, lung metastases, pulmonary metastasectomy, chemotherapy, meta-analysis

## Abstract

**Background:**

Because of the high rate of recurrence, the prognosis of patients with pancreatic ductal adenocarcinoma (PDAC) is still very poor despite underwent pancreatectomy and adjuvant chemotherapy. A few reports have suggested the feasibility and efficacy of surgical resection for pulmonary metastases of PDAC. However, the role of metastasectomy of recurrent PDAC remains controversial. The aim of this study is to evaluate the benefits of pulmonary metastasectomy in PDAC patients with lung metastases.

**Methods:**

We searched the PubMed, Embase, and Cochrane Library databases and extracted the hazard ratio (HR) with 95% confidence interval (CI) from eligible studies. Pooled HR with 95% CI were used to reveal the association between pulmonary metastasectomy and survival.

**Results:**

The meta-analysis encompassed data from nine studies, comprising 467 patients suffered PDAC with lung metastasis. The results (the pooled HR: 0.637, 95% CI: 0.531–0.764, *I*^2^ = 61.5%, *p* value = 0.008) indicated that patients with lung metastasis who underwent pulmonary metastasectomy seemed to have better survival when compared with patients who underwent only chemotherapy. The robustness of these pooled results was verified by our subgroup analysis and sensitivity analysis. Moreover, the varying sample sizes among studies contribute to the heterogeneity in the pooled hazard ratio (HR) for survival, as indicated by the meta-regression analysis (*p* value = 0.045).

**Conclusion:**

Pulmonary metastasectomy could prolong the survival in patients with lung metastases from PDAC. However, the present study is based on a relatively small number of patients and may include a selection bias. More multi-institutional prospective study is needed to evaluated the clinical value of pulmonary metastasectomy.

## Introduction

Pancreatic ductal adenocarcinoma (PDAC) is recognized as one of the most aggressive malignant digestive system tumors with a 5-year survival rate less than 10% (1). Because of a high recurrence rate after surgery, the prognosis of pancreatic cancer is dismal even after curative pancreatic resection. The recurrence patterns of PDAC are diverse, with lung metastases being one of the most common sites of distant spread (2, 3). The incidence rates of pulmonary metastases ranging from 2.9% to 21.8% have been reported (4–6). Patients with lung metastases from PDAC tend to have a relatively better prognosis compared to those with other types of hematogenous disseminations, such as liver or peritoneal metastases (7). Median overall survival (OS) after the initial treatment can be varied from 51 to 121 months in metachronous lung metastasis (8).

Chemotherapy is seemed to be the only therapeutic strategy for metastatic pancreatic cancer and surgical resection is generally not recommended. Nonetheless, some studies have shown that resection of the pulmonary metastases in patients with colorectal cancer could prolong survival time (9). In addition, some case reports or case series, as well as few retrospective studies have shown that pulmonary resection of isolated lung metastases is associated with long-term survival in some patients (4, 10, 11). However, the effect of surgical resection on extending the survival is still unclear because the selection of patients with relatively indolent diseases might cause survival benefits after pulmonary resection. Furthermore, oncological outcomes and clinical benefits of pulmonary resection for patients with lung metastases have not been clarified. The aim of our meta-analysis is to evaluate the clinical values of surgical management for lung metastases from PDAC by performing a detailed investigation of postoperative oncological outcomes after pulmonary resection.

## Materials and methods

This systematic review and meta-analysis was conducted following the Preferred Reporting Items for Systematic Reviews and Meta-Analyses (PRISMA) statement (12).

### Search strategies

PubMed, Embase, and Cochrane Library databases were searched for eligible articles up to September 1st, 2024. The search was conducted using medical subject headings (MeSH) in combination with free text words. The following search headings were used: “pancreatic cancer”, “lung”, “metastasis”, “recurrence”, “resection”, and “surgery”, and we used “AND”, “OR”, “NOT” for combination of these headings to avoid missing and wrong articles. The search strategy is described in the Supplementary Materials.

### Inclusion and exclusion criteria

All studies included in the meta-analysis were selected according to the following inclusion criteria: (1) all patients were diagnosed with PDAC with synchronous or metachronous lung metastases; (2) patients underwent the pulmonary resection; (3) survival data can be collected in the literature; (4) Newcastle-Ottawa Quality Assessment Scale (NOS) score ≥ 6. The exclusion criteria were as follows: (1) neuroendocrine tumor and other pathological types; (2) multiorgan metastases; (3) incomplete survival data; (4) abstracts, case reports, editorials, letters, systematic reviews, and comments; (5) overlapped or same population; and (6) duplicate studies.

### Data extraction

Two investigators (Pengcheng Zhao and Qiaoqi Jiang) independently extracted the necessary data from the included studies, and any disagreements were resolved by discussion until a consensus was reached. The following data were extracted from each study: first author, publication year, country, study design, number of patients, tumor site, number of lung metastases, follow-up duration, median survival time, and overall survival.

### Quality assessment

The Newcastle-Ottawa Quality Assessment Scale (NOS) was used to evaluate the quality of the included studies. Studies with a score of six or higher were considered high-quality studies (13). This work was also performed independently by two investigators (Pengcheng Zhao and Qiaoqi Jiang). Details of NOS score were showed in Supplementary Table S1.

### Statistical analysis

Stata 14.0 software was used for data analysis. The heterogeneity of the pooled effect was assessed using Cochran's *Q* test and the Higgins *I*^2^ statistic. *Q* test *p* value < 0.1 or *I*^2^ > 50% was considered significant heterogeneity, and a random-effect model was applied to estimate the pooled HR. While heterogeneity was not significant (*Q* test *p* value > 0.1 or *I*^2^ < 50%), a fixed-effect model was used. The graphical description of the statistical results was illustrated with forest plot. Sensitivity analysis was applied to reduce and explain heterogeneity among the studies. Furthermore, publication bias was visually checked through a funnel plot and then quantitatively analyzed using Begg's and Egger's tests. All statistical tests were two-sided, and a *p* value less than 0.05 were defined as statistically significant.

## Results

### Study selection

**Figure 1:**
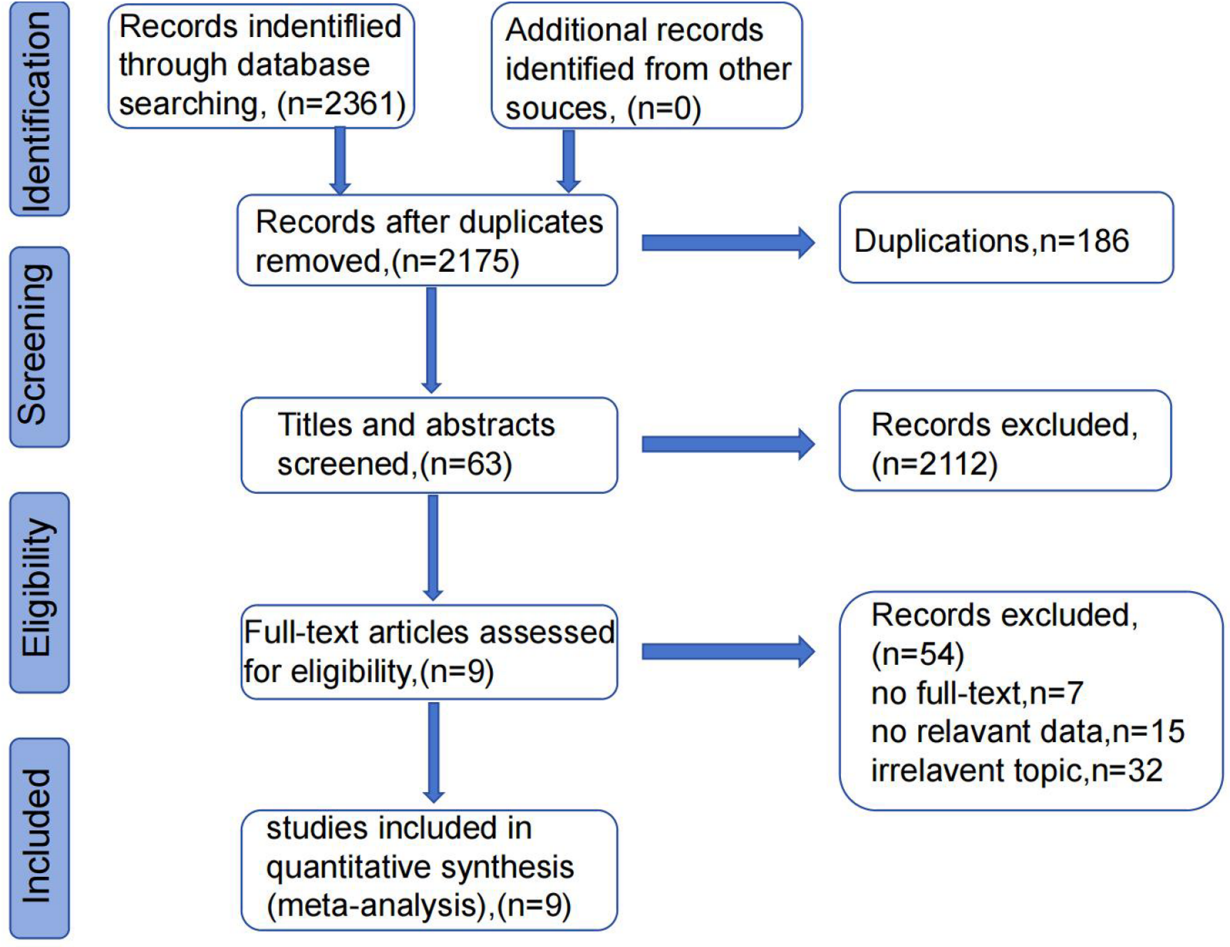
PRISMA flow diagram of eligible studies selection.

After the literature search, 2,361 articles were initially retrieved. After removing 186 duplicates, 2,175 articles remained. After screening the titles and abstracts, 2,112 articles were excluded for being irrelevant topics, reviews or meta-analysis, conference abstracts, letters, case reports, case series, or comments. Finally, 9 articles met our inclusion criteria, and 467 patients suffered PDAC with lung metastasis (10, 14–21). The detailed selection process was shown in Figure 1.

### Clinical characteristic of enrolled studies

All the studies were retrospective studies, and were mainly published in the past five years. The sample size of enrolled studies varied from 13 to 117, and a total of 467 patients suffered PDAC with synchronous or metachronous pulmonary metastases were enrolled. Among the included 467 patients, 189 patients underwent resection of lung metastases. In all selected articles, the median survival time from the initial pancreatectomy was reported for both the pulmonary metastasectomy group and the adjuvant chemotherapy group. The main characteristics of the included studies were presented in Table 1.

**Table 1:** The main characteristics of included studies.

Year	Author	Country	Age	Gender (M/F)	Patients, *n* = (no PM group)	Patients, *n* = (PM group)	Lung metastases (single/multiple)	RFI^a^	Chemotherapy (before PM)	Chemotherapy (after PM)	Follow-up (months)	PM group MST (months)	No PM group MST (months)
2023	Konishi	Japan	68 (37–83)	29/16	25	20	15/5^a^	33 (0–85)	4/20	12/20	NR	51	26
2023	Stuart	America	64.4	17/15	25	14	8/6^a^	24.9 ± 18.9	6/14	13/14	89.8 ± 31.0	52.7	42.6
2023	Takeda	Japan	70 (64–76)	21/13	20	14	18/16	NR	4/14	3/14	NR	52.7	23.8
2022	Homma	Japan	65.6 ± 11	58/59	85	32	24/8^a^	27.8 ± 15	7/32	24/32	NR	84	32.9
2022	Yun	Korea	64.5	39/44	68	15	23/60	NR	1/15	11/15	NR	26.6	19
2021	Mashiko	Japan	69 (55–80)	15/9	18	6	0/6^a^	24 (10.8–37.2)	0	2/6	44 (10–153)	50 (15.9–84.1)	37 (34.4–39.6)
2020	Shimizu	Japan	70.3 (59–81)	5/8	7	6	3/3^a^	26.1 (12–53)	0	4/6	NR	39 (28–76)	33 (14–40)
2019	Kim	Korea	NR	NR	8	15	NR	16	NR	NR	NR	36.5	9.5
2011	Arnaoutakis	America	69.4	14/17	22	9	NR	34 (21–49)	NR	NR	46	51	23

M, male; F, female; PM, pulmonary metastaectomy; RFI, recurrence-free interval from initial pancreatectomy to lung metastases; MST, median survival time; NR, not record.

^a^ PM group.

### Clinical benefits of lung metastasectomy in PDAC patients with lung metastases

**Figure 2:**
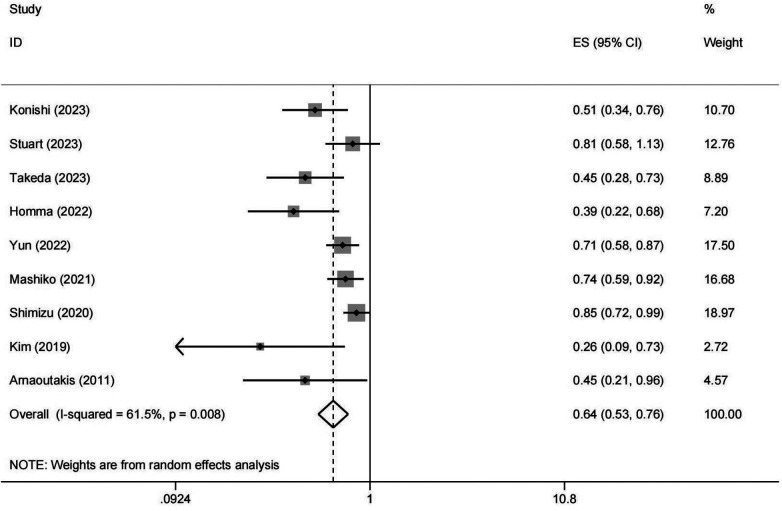
Forest plot of meta-analysis on OS between surgical and non-surgical groups. OS, overall survival; HR, hazard ratio; CI, confidence interval.

Nine studies investigated the resection of lung metastases in metastatic PDAC patients (10, 14–21). Given the inherent variability in study designs, patient populations, and treatment protocols in retrospective studies, significant heterogeneity was anticipated. As illustrated in Figure 2, the results (the pooled HR: 0.637, 95% CI: 0.531–0.764, *I*^2^ = 61.5%, *p* value = 0.008) indicated that resection of lung metastases could prolong the survival of the PDAC patients with pulmonary metastases.

### Sensitivity analysis and publication bias

**Figure 3:**
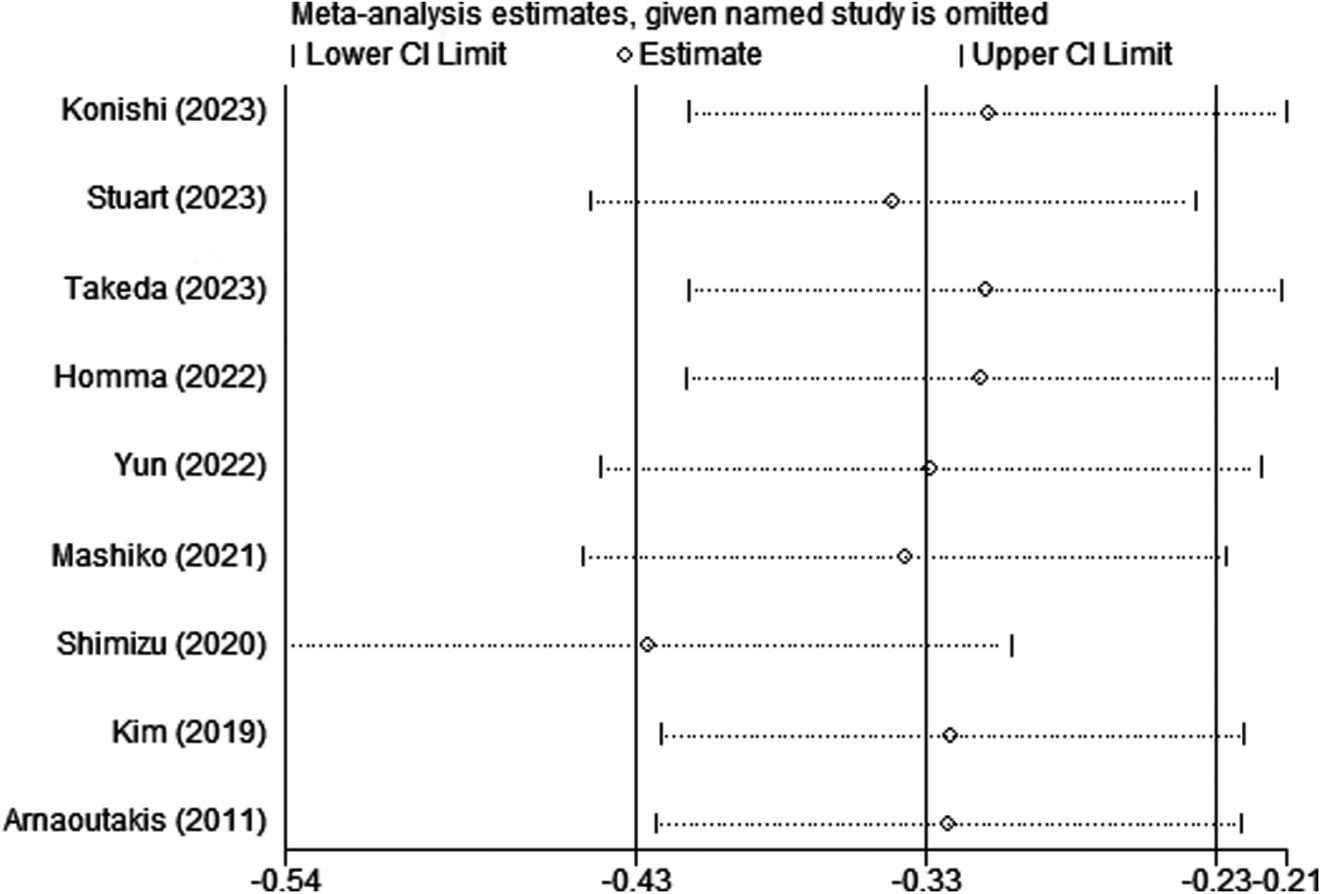
Sensitivity analysis of the surgical group. OS, overall survival.

**Figure 4:**
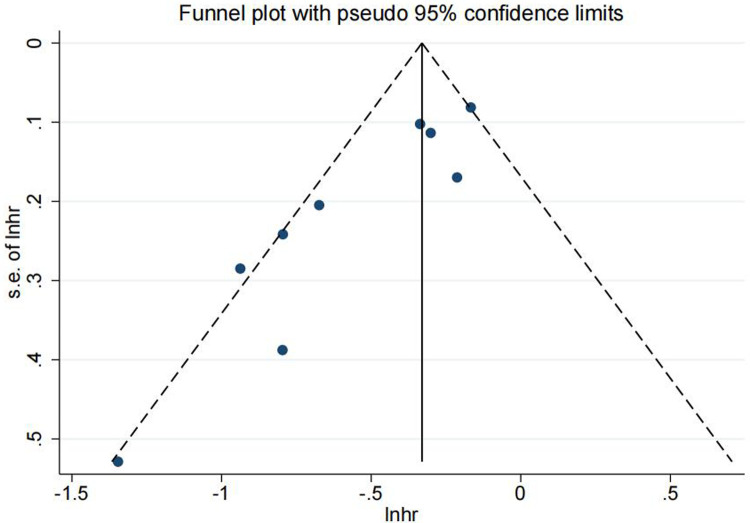
Funnel plot illustrating publication bias test result.

Sensitivity analysis was conducted to assess the effect of individual studies on the pooled HR, and the results suggested that omitting any individual study had no significant effect on the pooled HR (Figure 3). Furthermore, publication bias was investigated, Begg's test and Egger's test yielded *p*-values of 0.048 and 0.001, respectively. The funnel plot revealed that there was some extent of publication bias among included studies (Figure 4).

### Meta regression analysis and subgroup analyses

**Figure 5:**
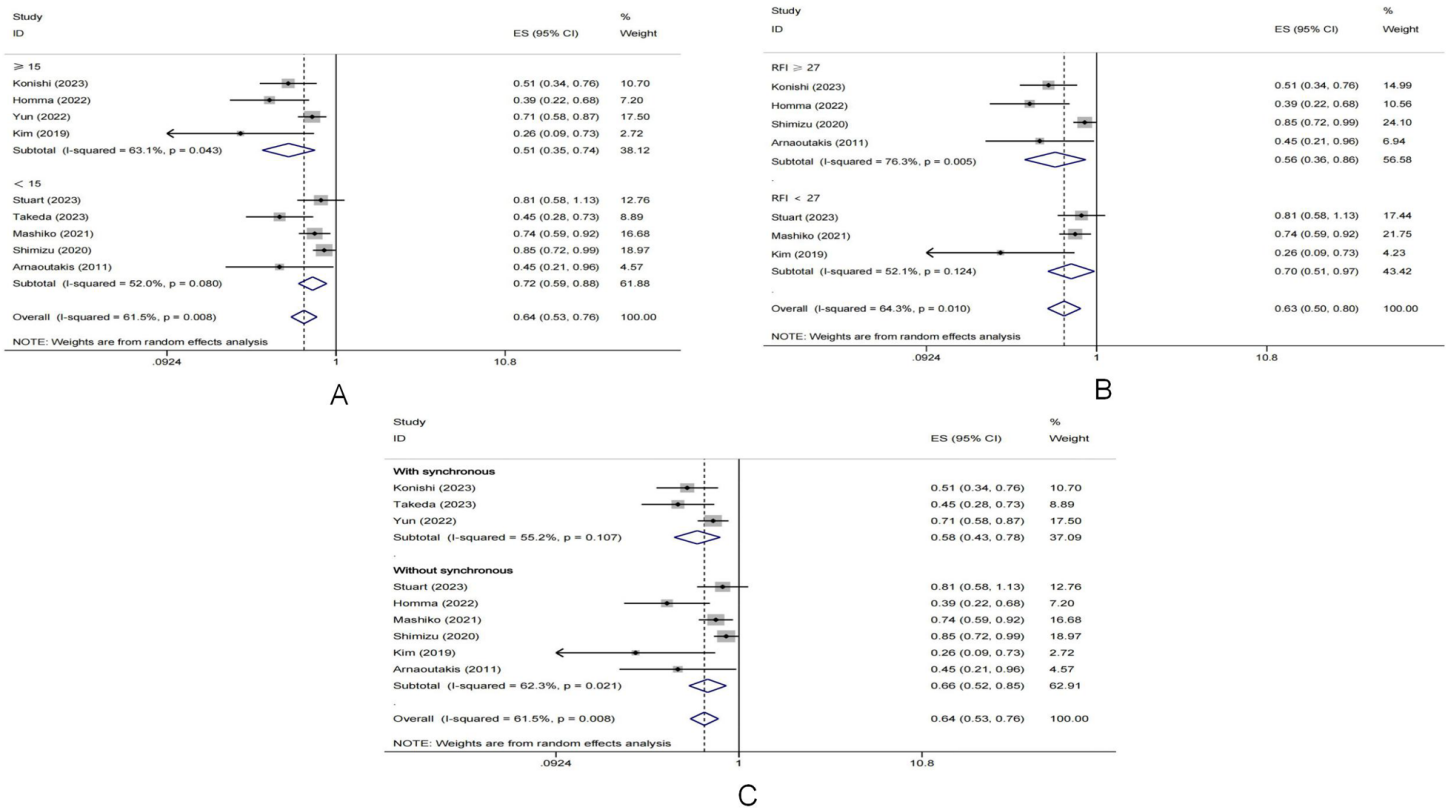
Forest plot of subgroup analyses based on **(A)** the number of patients underwent metastaectomy; **(B)** the length of RFI after primary pancreatectomy; **(C)** the timing of metastases.

To explore and explain the heterogeneity, meta regression analysis was applied. The *p* value of PM subgroup was 0.045. The results of meta regression indicated that the relatively small sample size might be a source of heterogeneity. In addition, subgroup analyses were conducted based on the number of patients in the PM group. The cutoff value was defined as 15, the pool HR in the subgroup with patients number fewer than 15 was 0.719 (95% CI: 0.589–0.878, *I*^2^ = 52.0%, *p* value = 0.08), while the results of another subgroup was: pooled HR = 0.513, 95% CI: 0.354–0.744, *I*^2^ = 63.1%, *p* value = 0.043. The results of subgroup analysis were shown in Figure 5A. Moreover, the results of another subgroup analyses based on the timing of lung metastases and the recurrence-free interval (RFI) from primary lesion pancreatectomy to lung metastases were also presented in Figures 5B,C.

## Discussion

Although surgical resection is the only treatment that offers long-term survival in selected patients with resectable pancreatic cancer, among patients who undergo pancreatectomy for PDAC, almost 80% patients develop locoregional and/or distant recurrence after primary pancreatectomy (22). The most common metastatic site is liver, followed by lung (2). Historically, palliative chemotherapy seemed to be the only option for PDAC patients with lung metastases. In recent years, pulmonary metastasectomy has been introduced as a novel treatment (23). We aimed to evaluate the benefits of pulmonary metastasectomy in PDAC patients with lung metastases.

In our meta-analysis, nine articles with 467 PDAC patients with synchronous or metachronous lung metastases were enrolled (10, 14–21). Our pooled analysis showed that pulmonary metastasectomy could significantly improve overall survival of PDAC patients with lung metastases compared to those patients who did not undergo resection of metastatic tumor (the pooled HR: 0.637, 95%CI: 0.531–0.764, I^2^ = 61.5%, *p* value = 0.008). Meta regression analysis was used to explore the heterogeneity. The *p* value was 0.045, less than 0.05, and the results indicated that the number of patients who underwent pulmonary metastasectomy might be the source of heterogeneity. Moreover, meta-regression analysis was also used based on the recurrence-free interval (RFI), and the *p* value was 0.627, suggesting that RFI may not be related to the heterogeneity. We believed that the the number of patients who underwent PM could have a significant impact on the survival, especially in the studies with a small number of patients. The smaller the sample size, the greater the heterogeneity of the results. The heterogeneity caused by publication bias could not be ignored either. In our study, Begg's test and Egger's test were used to evaluate the publication bias, and yielded *p*-values of 0.048 and 0.001, respectively. The results showed that there was a certain extent of publication bias in our study, which could impact the stability of the pooled results. To mitigate the impact of publication bias in future research, grey literature and unpublished studies should also be inculded. What's more, study design, patient population and treatment protocols might also be the potiential sources of heterogeneity. All included studies were retrospective, which inherently introduces variability in data collection methods and quality. Differences in study design, such as variations in patient selection criteria and the definition of lung metastases, may contribute to the observed heterogeneity. The characteristics of the patient populations varied across studies. Factors such as age, gender distribution, and tumor characteristics (e.g., synchronous vs. metachronous metastases) could influence survival outcomes. Variations in treatment protocols, including the type of pulmonary resection (e.g., wedge resection, segmentectomy, or lobectomy) and the use of adjuvant chemotherapy, could impact survival outcomes as well. The timing and duration of chemotherapy, both before and after pulmonary metastasectomy, may also differ across studies, contributing to the observed heterogeneity. To address this heterogeneity, we conducted subgroup analyses based on the number of patients undergoing pulmonary metastasectomy, the timing of lung metastases, and the RFI. These analyses provided insights into the potential sources of heterogeneity and helped identify subgroups where the benefits of pulmonary metastasectomy may be more pronounced.

Previous studies have revealed that the patients with pulmonary metastases often have a better prognosis than those with metastases at other sites, such as the liver. The prognosis of metastatic PDAC may be related to the duration of recurrence. It has been reported that late recurrence more commonly develops in the lung than in the liver or peritoneum (24). The definition of RFI was the interval between initial primary pancreatectomy and lung metastases. PDAC patients with a longer RFI could receive clinical benefits from pulmonary resection and have a better prognosis (10, 19). In Konishi's study (10), patients with an RFI of <28 months, as expected, had early tumor recurrence, resulting in continuous chemotherapy after lung metastases and poor prognosis, while patients with an RFI of ≥28 months did not have tumor recurrence within 12 months after pulmonary resection and had a longer chemotherapy-free interval. Univariate analyses also revealed that RFI from initial pancreatectomy to lung metastases of ≥28 months was associated with better disease-free survival (DFS) after pulmonary resection. Another study reported, in study of 15 cases, that patients who developed lung metastasis more than 17 months after initial pancreatectomy had a better prognosis compared to those who developed lung metastasis at an earlier time (25). Prolonged RFI from the initial pancreatectomy to the development of lung metastasis could be considered as a prognostic factor of PDAC patients with pulmonary metastases.

The number of lung metastases was also associated with the prognosis of patients. Patients with solitary metastases were more likely to undergo surgical resection and showed a longer median overall survival. In Homma's study, the results of multivariate analysis showed that solitary metastasis was identified as significant prognostic factor after lung resection (HR: 5.03; 95% CI: 1.195–21.144, *p* = 0.022) (16).

Oligometastases, defined as a state of limited number of metastases, were not equal to solitary metastases, and were considered as an intermediate state between solitary and multiple metastases. which may also benefit from surgical resection. Lung oligometastases were defined as having two to five lesions, based on previous reports on the local treatment of pulmonary metastatic lesions (26, 27). A study has reported that. patients with lung oligometastasis were more likely to undergo surgical resection (41% vs. 0%) and had a significantly better prognosis (41.3 vs. 17.6 months) than those with lung polymetastasis. Oligometastasis (HR, 0.43; 95% CI, 0.24–0.76) was identified as an independent factor predicting favourable OS in PDAC patients with distant metastasis confined to the lung (15). Moreover, the number and location of lung metastases would also determine the method of PM. The modalities for resection of lung metastases were wedge resection, segmentectomy and lobectomy. The type of pneumonectomy also had an effect on prognosis. In general, lobectomy was more thorough than wedge resection, but it also has a greater impact on lung function. Studies have shown that there is no significant difference in overall survival between lobectomy and wedge resection, but lobectomy may provide more complete tumor resection in some cases (28).

The role of chemotherapy was not ignored in the management of PDAC with pulmonary metastases equally. It was widely accepted that multidisciplinary treatment (MDT) was necessary for improving the prognosis of patients with advanced-stage pancreatic cancer (29, 30). In Yun's study, the results of multivariate analyses revealed that chemotherapy (HR = 0.434, *p* = 0.024), and chemotherapy cycles (HR = 0.300, *p* < 0.001) had significant effects on survival (17). One study also reported that 24 of 32 patients received adjuvant chemotherapy after pulmonary metastasectomy. The addition of postoperative chemotherapy after pulmonary resection significantly improved the time to recurrence to 41.5 months compared to the group without chemotherapy, and the median time from recurrence to death (RTD) was also longer in patients underwent postoperative chemotherapy (59.0 months vs. 7 months, *p* = 0.02). In the multivariate analysis, postoperative chemotherapy after metastasectomy (HR: 14.089; 95% CI: 1.729–114.77, *p* = 0.023) was identified as significant prognostic factors after lung resection (16). In summary, resection of lung metastases, especially lobectomy, combined with long RFI and adjuvant chemotherapy, could significantly improve overall survival of PDAC patients with pulmonary metastases.

While our meta-analysis demonstrates potential survival benefits of pulmonary metastasectomy in PDAC patients with lung metastases, it is crucial to consider the broader clinical context, including perioperative risks and postoperative complications. Patients with pancreatic ductal adenocarcinoma (PDAC) often present with significant systemic disease burdens, which may affect their tolerance to surgery. The decision to proceed with pulmonary metastasectomy should be carefully weighed against the potential risks and benefits for each individual patient. Pulmonary metastasectomy is associated with inherent surgical risks, including anesthesia-related complications, bleeding, infection, and respiratory complications. Given the compromised overall health status of many PDAC patients, these risks may be further exacerbated. Postoperative complications, such as prolonged hospital stays, respiratory failure, and delayed recovery, can significantly impact patient outcomes and quality of life. Therefore, a thorough preoperative assessment is essential to identify patients who are likely to benefit from surgery without undue risk. In addition, stereotactic body radiotherapy (SBRT) might be a promising alternative treatments for managing lung metastases in PDAC patients, offering high local control rates with minimal invasiveness. This approach may be particularly suitable for patients with significant comorbidities or those who are not surgical candidates due to poor performance status. Additionally, multidisciplinary collaboration in selecting patients for pulmonary metastasectomy was crucial for optimizing clinical outcomes.

This meta-analysis has several limitations. Firstly, all the included studies were retrospective. Retrospective studies are inherently prone to selection bias and recall bias, which may significantly influence the interpretation of our findings. Selection bias may arise from the non-randomized allocation of patients to surgical or non-surgical groups, potentially leading to an overestimation of the benefits of pulmonary metastasectomy. Additionally, the reliance on historical data may introduce recall bias, where the accuracy of data collection and reporting may vary between studies. These biases could affect the comparability between the surgical and non-surgical groups, and thus, the observed survival benefits attributed to pulmonary metastasectomy may not be entirely attributable to the intervention itself. Moreover, the retrospective design limits our ability to control for confounding variables that may influence survival outcomes, such as differences in patient demographics, tumor characteristics, and the quality of adjuvant therapies received. While we attempted to mitigate these biases through rigorous study selection criteria and quality assessment using the Newcastle-Ottawa Scale, the inherent limitations of retrospective data cannot be fully overcome. Secondly, the results of Begg's test and Egger's revealed that there was some extent publication bias among included studies. Thirdly, the sample size was relatively small, and large, multicenter RCTs are urgently needed. Moreover, The survival benefit observed in our analysis may not be uniform across all molecular subtypes of PDAC. For instance, patients with specific genetic mutations or biomarker expressions may respond differently to pulmonary metastasectomy. Future research should explore the role of molecular profiling in identifying patients who are most likely to benefit from surgical intervention.

## Conclusion

In summary, our meta-analysis revealed that PDAC patients with lung metastases who underwent pulmonary metastasectomy achieved longer survival compared with those who did not. Moreover, patients with long RFI and limited number of metastases are more likely to undergo metastasectomy and have a better survival. However, the retrospective nature of the included studies introduces significant limitations, including selection and recall biases, which may affect the validity of our findings. Therefore, caution should be exercised in interpreting these results. Prospective studies or randomized controlled trials are urgently needed to rigorously evaluate the clinical value of pulmonary metastasectomy in this patient population.

## Data Availability

The original contributions presented in the study are included in the article/Supplementary Material, further inquiries can be directed to the corresponding author.
