# Supplementary material for: The role of pulmonary metastasectomy in patients suffering pancreatic ductal adenocarcinoma with lung metastases: a systematic review and meta-analysis
Source: Front Surg. 2025 Feb 27;12:1535212. doi: 10.3389/fsurg.2025.1535212 (PMC11903735; doi:10.3389/fsurg.2025.1535212)
Supplement: Supplementary file 1 [file Table1.docx]

**Supplementary information**

**Search strategies in PubMed**

((("Pancreatic Neoplasms" [Mesh]) AND ("Pancreatic" [Title/Abstract] OR "pancreas"[Title/Abstract]) AND ("adenocarcinoma"[Title/Abstract] OR "carcinoma"[Title/Abstract] OR "cancer"[Title/Abstract] OR "neoplasm*"[Title/Abstract] OR "tumor"[Title/Abstract])) AND ((((lung[Title/Abstract]) OR (lungs[Title/Abstract])) OR (pulmonary[Title/Abstract])) AND ((((((metastasis[Title/Abstract]) OR (metastases[Title/Abstract])) OR (metastatic[Title/Abstract])) OR (oligometastasis[Title/Abstract])) OR (oligometastatic[Title/Abstract])) OR (oligometastases[Title/Abstract])))) AND (((((((((resection[Title/Abstract]) OR (resections[Title/Abstract])) OR (surgery[Title/Abstract])) OR (surgeries[Title/Abstract])) OR (operation[Title/Abstract])) OR (operations[Title/Abstract])) OR (pneumonectomy[Title/Abstract])) OR (pulmonectomy[Title/Abstract])) OR (metastasectomy[Title/Abstract]))

**Table S1.** The Newcastle-Ottawa scale for quality assessment of include studies.

| **Study** | **Selection** | | | | **Comparability** | **Outcome** | | | **Total score** |
| --- | --- | --- | --- | --- | --- | --- | --- | --- | --- |
|  | Representative-ness  of the exposed  cohort | Selection  of the  non-exposed  cohort | Ascertainment  of exposure | Demonstration  that outcome  of interest was  not present at  start of study | Comparability  of cohorts on  the basis of  the design or  analysis | Assessment  of outcome | Was follow-  up long  enough for  outcomes to occur | Adequacy  of follow  up of  cohorts |  |
| **Total score** | 1 | 1 | 1 | 1 | 2 | 1 | 1 | 1 | 9 |
| Konishi | 1 | 1 | 1 | 1 | 2 | 1 | 0 | 0 | 7 |
| Stuart | 1 | 1 | 1 | 1 | 0 | 1 | 1 | 0 | 6 |
| Takeda | 1 | 1 | 1 | 1 | 1 | 1 | 1 | 0 | 7 |
| Homma | 1 | 1 | 1 | 1 | 1 | 1 | 1 | 0 | 7 |
| Yun | 1 | 1 | 1 | 1 | 0 | 1 | 1 | 0 | 6 |
| Mashiko | 1 | 1 | 1 | 1 | 1 | 1 | 1 | 1 | 8 |
| Shimizu | 1 | 1 | 1 | 1 | 0 | 1 | 1 | 1 | 7 |
| Kim | 1 | 1 | 1 | 1 | 0 | 1 | 1 | 1 | 7 |
| Arnaoutakis | 1 | 1 | 1 | 1 | 0 | 1 | 1 | 0 | 6 |
